# The Effect of the Addition of Silicon Dioxide Particles on the Tribological Performance of Vegetable Oils in HCT600X+Z/145Cr46 Steel Contacts in the Deep-Drawing Process

**DOI:** 10.3390/ma18010073

**Published:** 2024-12-27

**Authors:** Tomasz Trzepieciński, Krzysztof Szwajka, Marek Szewczyk, Joanna Zielińska-Szwajka, Ján Slota, Ľuboš Kaščák

**Affiliations:** 1 Department of Manufacturing Processes and Production Engineering, Faculty of Mechanical Engineering and Aeronautics, Rzeszów University of Technology, al. Powstańców Warszawy 8, 35-959 Rzeszów, Poland; 2 Department of Integrated Design and Tribology Systems, Faculty of Mechanics and Technology, Rzeszów University of Technology, ul. Kwiatkowskiego 4, 37-450 Stalowa Wola, Poland; kszwajka@prz.edu.pl (K.S.); m.szewczyk@prz.edu.pl (M.S.); 3 Department of Component Manufacturing and Production Organization, Faculty of Mechanics and Technology, Rzeszów University of Technology, ul. Kwiatkowskiego 4, 37-450 Stalowa Wola, Poland; j.zielinska@prz.edu.pl; 4 Institute of Technology and Materials Engineering, Technical University of Košice, Mäsiarska 74, 040 01 Košice, Slovakia; jan.slota@tuke.sk (J.S.); lubos.kascak@tuke.sk (Ľ.K.)

**Keywords:** deep-drawing, metal forming processes, sheet metal forming, silicon dioxide, SiO_2_ particles

## Abstract

Friction is an unfavourable phenomenon in deep-drawing forming processes because it hinders the deformation processes and causes deterioration of the surface quality of drawpieces. One way to reduce the unfavourable effect of friction in deep-drawing processes is to use lubricants with the addition of hard particles. For this reason, this article presents the results of friction tests of dual-phase HCT600X+Z steel sheets using the flat die strip drawing test. Sunflower oil and rapeseed oil with the addition of 1, 5 and 10 wt.% of silicon dioxide (SiO_2_) particles were used as lubricants. Tests were also carried out in dry friction conditions and lubricated conditions using SiO_2_-modified oils and oils without the addition of particles, as a reference. Tests were carried out at different pressure values between 2 and 8 MPa. The effect of friction on the change in sheet surface roughness was also examined. For the entire range of pressures analysed, pure sunflower oil showed lower efficiency in reducing the coefficient of friction compared to pure rapeseed oil. In the pressure range of 4–8 MPa, the lubricants with 5 wt.% and 10 wt.% of particles were more effective in reducing friction than the biolubricant with the addition of 1 wt.% of SiO_2_. The lowest average roughness was observed for lubrication with sunflower oil containing 5 wt.% of particles. In relation to rapeseed oil, the addition of 10 wt.% of SiO_2_ provided a sheet surface with the lowest average roughness.

## 1. Introduction

Deep-drawing is one of the methods classified as a sheet metal forming process, which can be used to obtain thin-walled products of a complex shape [1,2]. Conventionally, deep-drawing is performed using tools consisting of a die, a punch and a blankholder. During forming, the sheet metal experiences a complex state of stress. Tensile stresses dominate in the side wall of the drawpiece. Meanwhile, in the flange area, the sheet metal is subjected to compressive stresses in the thickness direction. The complex state of stress and deformation occurring in deep-drawing means that different tribological tests are used to determine the coefficient of friction (CoF) in specific areas of the formed sheet metal [3]. The strip drawing test (SDT), which was used in this article to evaluate the CoF, models the friction conditions in the blankholder action zone [4].

The modification of friction conditions in deep-drawing processes can be achieved by using appropriate anti-wear coatings on the tools [5], texturing the tool surface [6] and using effective lubricants [7]. The last of these methods consists of modifying the properties of the lubricants by adding hard particles, usually metal oxides such as SiO_2_, TiO_2_, Al_2_O_3_, ZnO and CuO [8,9]. These additives act as a third body that separates the rubbing surfaces from metallic contact [10]. In addition to metal oxides, the tribological performance of the following additive particles or nanoparticles (NPs) has been studied over the years: MoS_2_, WS_2_, hBN, MoS_2_, graphite, PbS, H_3_BO_3_, CaCO_3_, halloysite clay nanotubes and magnesium stearate [11,12]. TiO_2_/SiO_2_ NPs are also used in emulsions to stabilise water droplets in oil. By increasing the mass fraction, the NPs accumulate on the surface of the droplets and increase their stability up to the saturation point. The results of studies on the stability of oil droplet dispersion and the lubrication efficiency of emulsions containing TiO_2_/SiO_2_ NPs presented in [13] show that there is a minimum amount of NPs required to ensure stable droplet dispersion.

In the last two decades, much emphasis has been placed on ecological aspects in industrial practice, mainly by reducing the use of oils produced from crude oil [14,15]. The environmental sustainability of sheet metal forming processes, especially in the automotive industry, is currently considered a priority for an eco-friendly economy. Sunflower, coconut, soybean, peanut, linseed, rapeseed and many other oils are considered potential biolubricants [16]. Vegetable oils contain triglycerides of fatty acids and polar compounds [17] and have a strong tendency to adsorb on metal surfaces, thus providing good lubricity under boundary lubrication conditions [18]. One approach to reducing friction under boundary lubrication conditions is the use of dilute solutions of fatty acids (i.e., oleic, linoleic and stearic) in sunflower oil [16]. Oleic, linoleic and stearic acids contain a chain of 18 carbons, to avoid any influence of chain length on lubricant performance [16]. Due to the long-chain fatty acids, the structure of vegetable oil is amphiphilic. Vegetable oils are suitable as a base for the easy preparation of lubricants with NP additives for use in both boundary and hydrodynamic lubrication conditions [19,20,21]. Vegetable oils are an alternative to conventional lubricants due to their high load-bearing capacity, low evaporation rates, high flash point, good anti-wear properties, high viscosity index and non-toxicity [22]. Non-edible bio-oils have the potential as applications as lubricants due to their high fatty-acid content (60–80%) [18,19].

Silica dioxide NPs are widely used in various applications due to their very good potential for creating a stable suspension and homogenous size. Seibert et al. [23] investigated the effect of using SiO_2_ NPs (0.2 wt.%) on the tribological properties of 10W-40 engine oil using the ball-on-disc test. They found that chemical functionalisation is the most satisfactory method for creating a stable suspension. Sankar and Duraivelu [24] blended SiO_2_-Al_2_O_3_-TiO_2_ NPs in SN 500 base oil. They tested the friction performance of suspensions using the pin-on-disc test under varying load and speed conditions. Mahara and Singh [25] analysed SiO_2_ NPs as neem oil additives (0.15–0.9 wt.%). They found, using the pin-on-disc test, that adding NPs up to 0.3% to the neem oil provided better lubricity of the oil. Peng et al. [26] examined the effect of the size of SiO_2_ NPs added to paraffin oil (0.0125–4 wt.%). The results of the ball-on-ring test revealed that surface-modified SiO_2_ NPs with oleic acid having a diameter of 58 nm provided better friction reduction than pure liquid paraffin. Li et al. [27] dispersed 0.3 wt.% of SiO_2_ NPs in ST5W/30 mobile oil. Using a reciprocating tribotester, they found the coefficient of friction (CoF) decreased relative to pure ST5W/30 gas mobile oil. Spinache et al. [28] found that the addition of 2 wt.% of SiO_2_ NPs into the engine oil decreased the internal friction torque by 9% in comparison to that of engine oil without the addition of NPs. Prabu et al. [29] investigated the tribological characteristics of synthesised gear oil with the addition of SiO_2_ NPs. Using a four-ball tribotester, they found that the addition of 0.4 wt.% of SiO_2_ NPs significantly reduced the wear depth and wear scar diameter. Cortes et al. [30] studied the tribological performance of sunflower oil modified with silicon dioxide SiO_2_ NPs as lubricant additives at concentrations between 0.25 and 1.25 wt.%. The results of block-on-ring sliding tests showed that the CoF decreased with the addition of SiO_2_ NPs by 93.7% when compared to non-modified sunflower oil. Xie et al. [31] tested EOT5 neat lubricant oil with the addition of SiO_2_ NPs in the range of 0.1–1 wt.%. A linear reciprocating ball-on-disc was used to determine the CoF of AZ31 magnesium alloy sheets. It was found that nanolubricants effectively improved the surface quality of sheet metals during the cold rolling process. Pang et al. [32] investigated vegetable oil/SiO_2_ (0.5 wt.%) nanolubricants using ring compression tests. The formulated lubricant exhibited better tribological performance than that of the base oil. Cortes and Ortega [33] prepared lubricants by adding SiO_2_ and CuO NPs (0.25–1.25 wt.%) to coconut oil. A block-on-cylinder tribotester was used to evaluate the lubricants. The addition of SiO_2_ NPs decreased the CoF by 93.25% compared to coconut oil without NPs. According to Taha-Tijerina et al. [34], the tribological evaluations showed that the addition of SiO_2_ resulted in a significant improvement in the load-carrying capacity. For instance, at 0.25 wt.%, enhancements of 60% were observed for sunflower oil.

There are limited studies in the literature on the use of vegetable-based lubricants with the addition of SiO_2_ in sheet metal forming. Usually, the studies available in the literature are devoted to reducing the CoF in bulk plastic working processes. The number of works on the use of NPs in sheet metal forming is very limited. To the best of the authors’ knowledge, tribological studies of biolubricants with the addition of SiO_2_ to HCT600X+Z/145Cr46 steel contacts are not available. A 145Cr6 cold-work tool steel is the basic material used for deep-drawing stamping dies operating at ambient temperature. This article presents the results of friction tests of dual-phase HCT600X+Z steel sheets in a flat die SDT. Sunflower oil and rapeseed oil with silicon dioxide (SiO_2_) particles added at 1, 5 and 10 wt.% were used as lubricants. The tribological tests were carried out at different pressure values.

## 2. Materials and Methods

### 2.1. Test Material

Friction tests were carried out for 0.8 mm thick dual-phase HCT600X+Z steel sheets produced in a hot rolling process. This steel is also denoted as DP600+Z or 1.0941. The main elements in this steel, apart from iron, are manganese (1.89 wt.%), silicon (0.26 wt.%), aluminium (0.026 wt.%) and carbon (0.09 wt.%) [35]. The content of other elements is less than 0.1 wt.%. HCT600X+Z steel is mainly used in the automotive industry due to its advantageous mechanical properties. A low yield strength to tensile strength ratio provides excellent ductility during cold forming. Dual-phase (DP) steels have a high tendency to harden due to their microstructure. DP steels consist mainly of a ferritic matrix surrounded by harder martensite islands. During the forming of DP steel sheets, the deformation is concentrated in the lower-strength ferrite. The high susceptibility of these steels to mechanical joining [36], electric welding and laser welding, and the MAG method [37], combined with good formability [38], are the basic premises for using these steels in the automotive industry.

**Figure 1:**
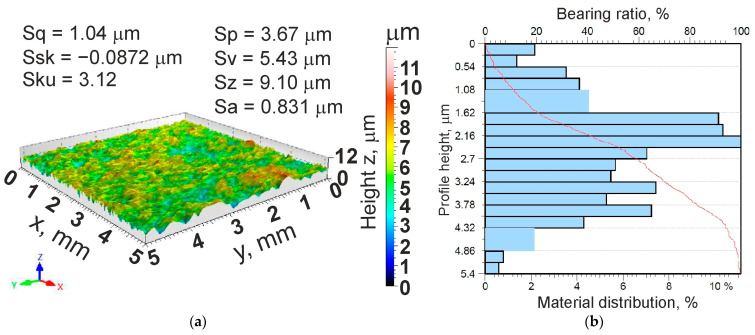
(**a**) Surface topography and (**b**) material ratio curve of an HCT600X+Z steel sheet.

The basic mechanical properties of HCT600X+Z steel sheets were determined in a uniaxial tensile test using a TIRAtest 2300 universal testing machine (TIRA GmbH, Schalkau, Germany). Test samples (Figure 1) were cut at an angle of 0°, 45° and 90° to the rolling direction—in this way, the average values of the mechanical parameters were determined (Table 1). The average hardness of the sheet material measured with a Qness 60 EVO hardness tester (Qness, Mammelzen, Germany) was 206 HV.

Surface roughness is a basic parameter characterising the surface of sheet metal intended for deep-drawing processes. Figure 1a,b show the surface topography and the material ratio curve, respectively. A Hommel-Etamic T8000RC (Jenoptik, Jena, Germany) profilometer was used to determine the surface roughness parameters in accordance with ISO 25178-2 [39]. 

### 2.2. Experimental Procedure

**Figure 2:**
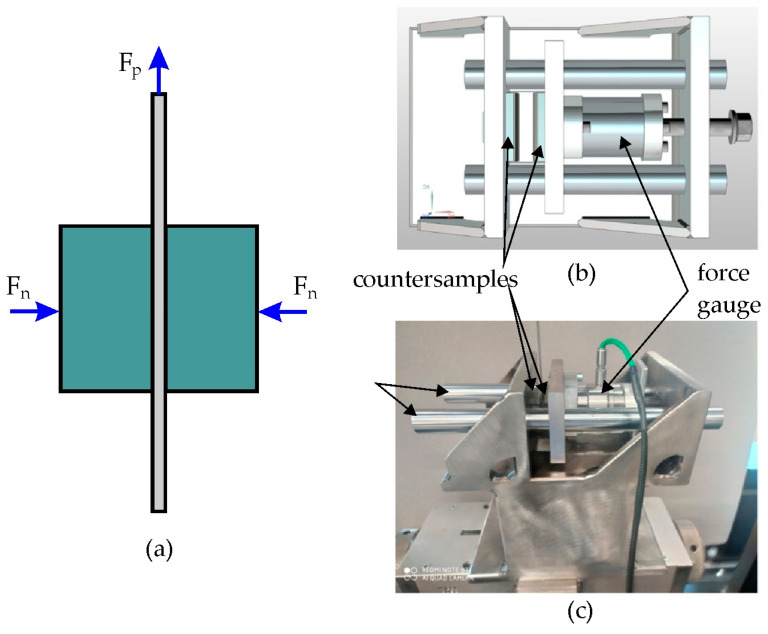
(**a**) Scheme of the SDT, (**b**) model and (**c**) photograph of the SDT tribometer.

**Figure 3:**
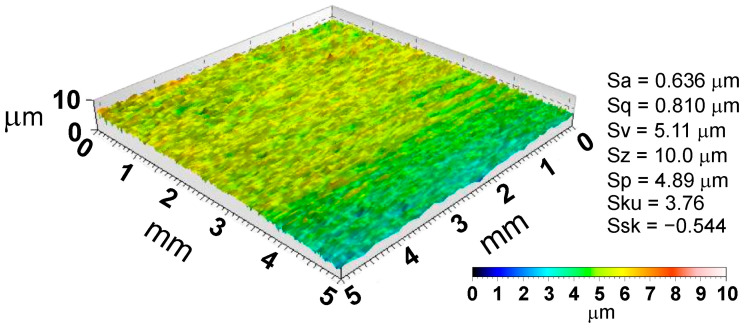
Surface topography of countersamples and their basic surface roughness parameters.

The SDT is a basic tribological test in sheet metal forming and consists of pulling a strip of sheet metal between flat countersamples (Figure 2a). The tester constructed by the authors consists of a body with guide bars on which horizontally moving countersamples roll on the flat work surface (Figure 2b,c). Countersamples were made of cold-worked tool steel 145Cr46 (C = 1.4–1.6 wt.%; Mn = 0.5–0.7 wt.%; Cr = 1.3–1.5 wt.%; Si = 0.15–0.3 wt.%; S, P ≤ 0.0035, Fe—balance). The average hardness of the countersample surface measured with a Qness 60 EVO hardness tester under a load of 98.07 N was 197.2 HV. The surface topography of the countersamples and the basic roughness parameters measured using a T8000RC profilometer (Jenoptik, Jena, Germany) are shown in Figure 3.

After placing the strip sample between the working surfaces of the countersamples, they are pressed together with a specified force. Taking into account the width of the countersamples and their working area, the pressing forces in the tests were set to obtain contact pressures equal to 2, 4, 6 and 8 MPa. To obtain these contact pressure values, the following normal forces were used: 1 kN, 2 kN, 3 kN and 4 kN, respectively. The nominal contact area of each countersample with the sheet metal surface was 500 mm^2^. Pressures of this value occur in the zone where the blankholder acts in deep-drawing processes [15,40]. Sample strips of sheet metal with a width of 25 mm and a length of about 140 mm were used.

**Figure 4:**
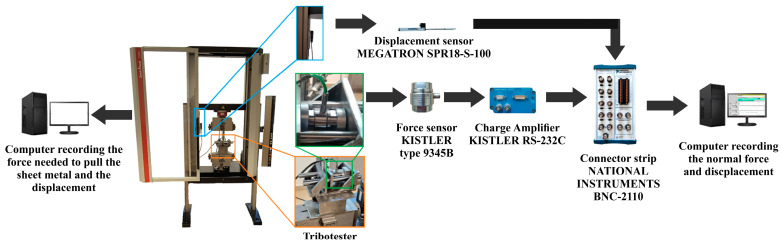
Schematic diagram of the test stand.

**Figure 5:**
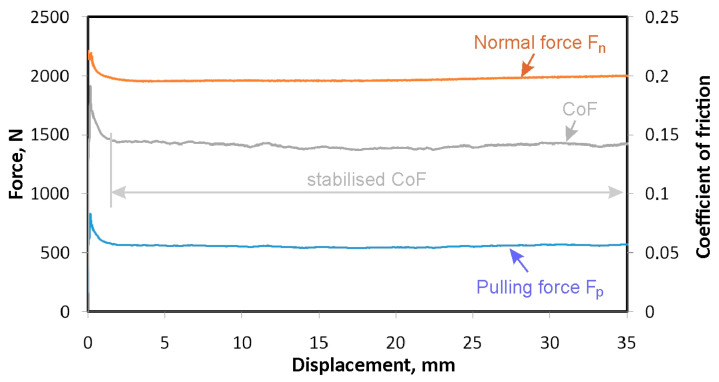
The variation in the force parameters and CoF during testing of HCT600X+Z sheet metal under the following conditions: lubrication with sunflower oil, contact pressure of 4 MPa.

One end of the strip sample was fixed in the upper grip of the Zwick/Roell Z100 tensile testing machine (Zwick/Roell, Ulm, Germany). The body of the device was fixed in the lower grip of this machine. After setting the appropriate force F_n_ (Figure 2a) measured by a Kistler^®^ type 9345B force sensor (Kistler, Winterthur, Switzerland), the upper grip of the testing machine was set in motion. The pulling force of the sample F_p_ was measured using the measuring system of the testing machine. The force F_n_ was recorded by means of software coupled with a BNC-2110-shielded connector block (National Instruments, Austin, TX, USA) (Figure 4). The measurement frequency of both forces was 100 Hz. After correlating the forces F_n_ and F_p_, the variation in the forces F_n_ and F_p_ (Figure 5) was determined during each test. The displacement of the sample during testing was measured using a SPR18-S100 (Elektronik GmbH & Co. KG, Putzbrunn, Germany) potentiometric linear transducer. The variation in normal force and sample displacement through the BNC-2110 terminal block (National Instruments, Austin, TX, USA) were registered using a personal computer. On this basis, the coefficient of friction was determined according to the relationship [41,42]:

(1) $$\mu =\frac{F_{p}}{{2F}_{n}}$$

Then, the average CoF was determined based on three repetitions. Unrefined sunflower (Skolej S.C., Koscielec, Poland) and rapeseed oils (Skolej S.C., Koscielec, Poland) were used in the tests. The viscosity at 23 °C of sunflower and rapeseed oils determined using a glass capillary viscometer according to the ISO 3105 [43] was 58 mm^2^/s and 51 mm^2^/s, respectively. In this study, we used the viscosity value of the base oils. Yalçın et al. [44] found that there was no significant viscosity change using different particle sizes of SiO_2_ at the same concentration. Other research [45,46] also obtained similar conclusions in terms of the effects of particle concentration on oil viscosity. However, according to Fedele et al. [47], a small increase in NP concentration resulted in increased viscosity of the suspension.

**Figure 6:**
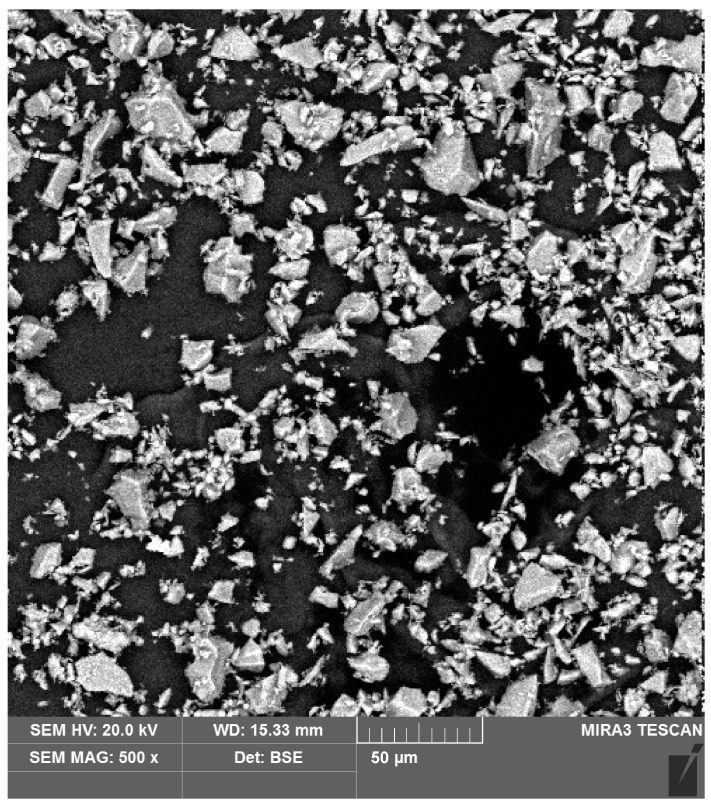
SEM micrograph of SiO_2_ particles.

In order to check the effect of individual oils and additives on friction conditions, a series of tests were carried out under dry friction and lubricated conditions with pure and modified vegetable oils. The base for the lubricants was sunflower and rapeseed oils, to which SiO_2_ powder was added in proportions of 1, 5 and 10 wt.%. The size of the SiO_2_ particles was from several micrometres to about 35 μm (Figure 6). However, the size of the major fraction particles was between 5 and 15 μm. Most studies of modified lubricants concern nano-sized particle additives. Due to the fact that the surface roughness of the sheets used in deep-drawing processes is of the order of micrometres and not nanometres, we intentionally used relatively large SiO_2_ particles. With large particles, it is easier to obtain the rolling effect of the particles between the contact surfaces.

In order to obtain the appropriate content of SiO_2_ powder in the oils, a CPA225D-0CE (Sartorius Weighing Technology GmbH, Goettingen, Germany) precision scale analytical balance was used. Then, using a mechanical stirrer, the prepared oils with SiO_2_ powder added were mixed, obtaining a uniform suspension. The mixing process itself lasted about 15 min. After the mixing process was completed, the friction process was carried out immediately. Particles do not dissolve in the tested oils. Oil is a carrier of particles, which are delivered in it to the contact zone. As a third body, the task of the particles is to limit the metallic contact of the sheet metal surface and the tool. Based on preliminary studies, we found that 15 min of mixing is sufficient to thoroughly mix the particles in the oils. Mechanical stirring usually takes 15–30 min [48,49]. In order to ensure the repeatability of the tests and eliminate the influence of suspending capacity on the friction test results, we conducted all tests immediately after mixing the oil.

Thirty-six different tests (Table 2) were performed with three repetitions. In this way, the average value of the CoF was determined for each of the planned measurements.

## 3. Results and Discussion

### 3.1. Coefficient of Friction

**Figure 7:**
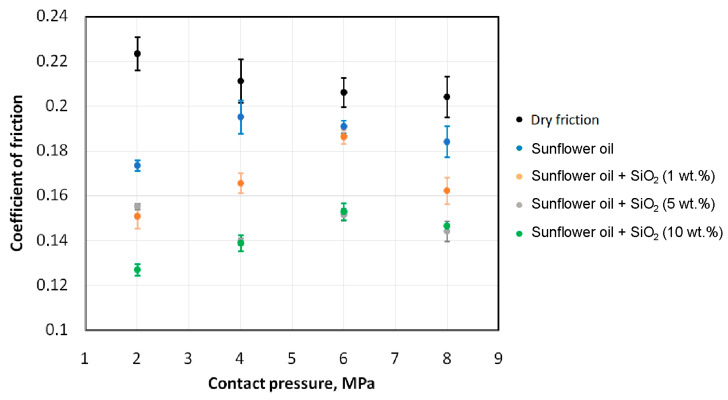
Effect of contact pressure on the CoF of HCT600X+Z steel sheets lubricated with sunflower oil.

**Figure 8:**
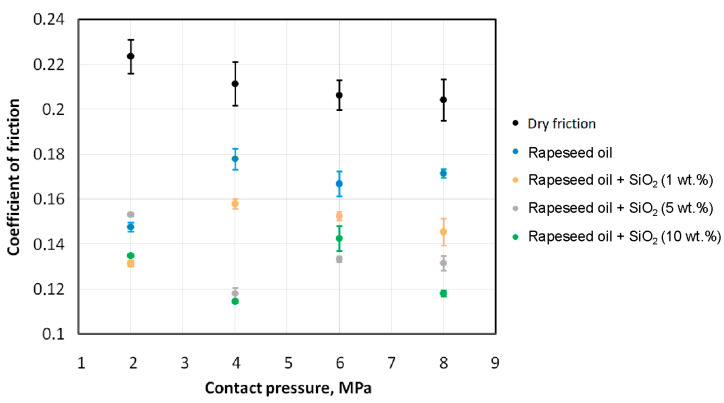
Effect of contact pressure on the CoF of HCT600X+Z steel sheets lubricated with rapeseed oil.

Figure 7 and Figure 8 present the effect of contact pressure on the coefficient of friction of HCT600X+Z steel sheets lubricated with sunflower oil and rapeseed oil, respectively. During dry friction, a tendency for the coefficient of friction to decrease with increasing contact pressure was observed. Such a relationship was also observed by Sigvant et al. [50] and Vollertsen and Hu [51]. This phenomenon can be explained by the lack of proportionality between friction force and normal force in the absence of lubricant, when metallic contact between the surfaces in contact dominates [50,51]. Dou and Xia [52] explained the decreasing CoF with increasing contact pressure by the principle that the real contact area increases with increasing normal load. Since the magnitude of the increase in pulling force F_p_ in Equation (1) is smaller than the increase in the normal load, the CoF decreases with increasing normal load F_n_.

**Figure 9:**
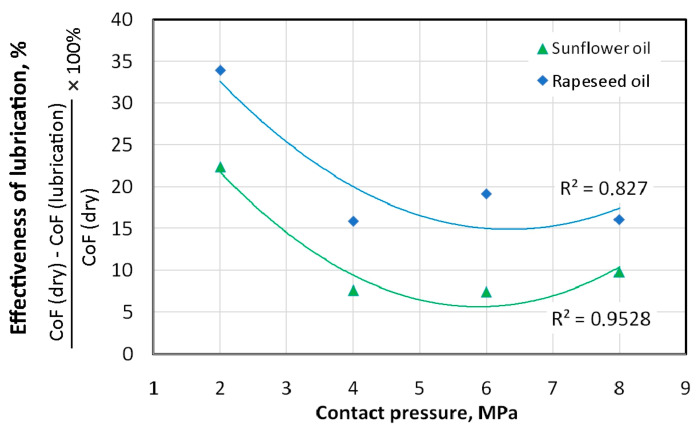
Effect of the type of non-modified lubricant on the effectiveness of the lubrication.

Unmodified (pure) sunflower oil showed lower efficiency in reducing the coefficient of friction compared to unmodified rapeseed oil. This relationship is valid for all the analysed contact pressures. The highest lubrication efficiency of unmodified oils was observed at the lowest analysed pressure (2 MPa). This efficiency for sunflower and rapeseed oils was 26.7% and 37.7%, respectively (Figure 9). For higher analysed contact pressures, lubrication efficiency decreased rapidly and amounted to about 17% for rapeseed oil and 9% for sunflower oil. With increasing contact pressure, the intensity of the mechanical interaction of the asperity summits increases, and the lubricant is not able to effectively reduce friction. Under contact pressure conditions of 2 MPa, the effect on the CoF of the amount of SiO_2_ particles added is not unequivocal. The greatest CoF reduction was provided by sunflower oil with 10 wt.% of SiO_2_ added. However, this oil with 1 wt.% of SiO_2_ added showed better performance than oil containing 5 wt.% of SiO_2_ (Figure 7). In the pressure range of 4–8 MPa, lubricants with SiO_2_ added ensured a reduction in the coefficient of friction, compared to dry friction conditions (Figure 7 and Figure 8). In addition, under these contact pressure conditions, the lubricants with the addition of 5 wt.% and 10 wt.% of SiO_2_ particles were more effective in reducing friction than the lubricant containing 1 wt.% of SiO_2_. These lubricants reduced the coefficient of friction to almost the same extent between 26.5% (6 MPa) and 34.9% (4 MPa), relative to dry friction (Figure 7). In the contact pressure range between 2 and 6 MPa, the rapeseed oil with 5 wt.% of SiO_2_ particles provided a reduction in the coefficient of friction of between 35.3% (6 MPa) and 45.0% (4 MPa). The coefficient of friction during the tests with rapeseed oil with 10 wt.% of SiO_2_ was reduced between 30.9% (6 MPa) and 46.7% (4 MPa). In summary, the effectiveness of the rapeseed oil-based lubricants is dependent on the contact pressure.

As noted in [30] with the increase in SiO_2_ content in sunflower oil, the viscosity of the lubricant increases, which in turn increases the resistance of the lubricant film to contact pressures. In this way, it is possible to explain the small changes in the coefficient of friction with the increase in pressure from 4 MPa to 8 MPa (Figure 7). It should also be noted that the SiO_2_-based lubricant is characterised by shear thinning behaviour [30,53].

**Figure 10:**
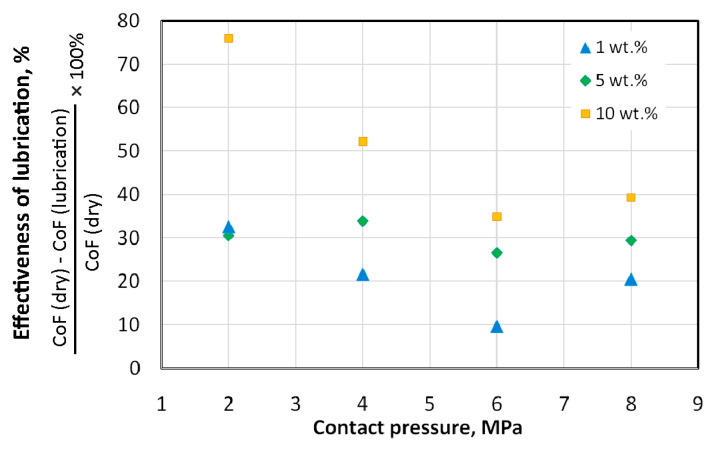
Effect of the addition of SiO_2_ on the effectiveness of lubrication with sunflower oil.

**Figure 11:**
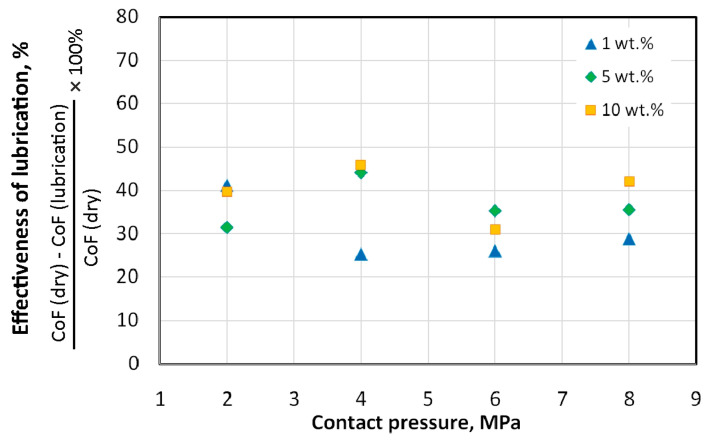
Effect of the addition of SiO_2_ on the effectiveness of lubrication with rapeseed oil.

The lubrication efficiency of oils with added SiO_2_ particles is shown in Figure 10 and Figure 11. For sunflower oil (Figure 10), the highest efficiency was demonstrated by sunflower oil with 10 wt.% of SiO_2_ added. During friction under contact pressure of 2 MPa, the oil with 10 wt.% of SiO_2_ added was more than twice as effective as the other oils. After exceeding the contact pressure of 2 MPa, there is a clear tendency for the lubrication efficiency to increase with increasing SiO_2_ content in the oil (Figure 10). The lubrication efficiency of modified rapeseed oil is more uniform for the entire range of analysed contact pressures (Figure 11) in comparison to lubrication with modified sunflower oil (Figure 10). The lowest efficiency (about 25%) was recorded during friction with sunflower oil modified with 1 wt.% of SiO_2_ content. The highest effectiveness of lubrication does not exceed 47%.

The beneficial effect of using hard particles as additives in lubricants can be explained by several lubrication mechanisms. The first is the mending effect, which is when the cavities in the surface topography are filled by the particles [54]. The polishing effect is when the particles take part in flattening the summits of the asperities of the rubbing bodies [26,55]. The particles can also interact with the rubbing surfaces to form protective films [56]. The last mechanism is the rolling effect, which occurs when the particles are the bodies separating the rubbing surfaces [57].

Xie et al. [58] also observed the beneficial effects of a lubricant with added SiO_2_ compared to the EOT5 base oil. The optimum weight concentration was 0.7 wt.%. It should be noted that the optimum concentration of particles depends on their size, the base oil properties, the roughness of rubbing surfaces and the contact pressures [32]. Tan et al. [59] investigated different concentrations of SiO_2_ (0.5–2.5 wt.%, size 5–15 nm) in lubricants containing extreme pressure (EP) additives. Based on the iconic experiments of ultra-high strength steel sheets, the optimal concentration was found to be 2 wt.%. Analysis of the literature indicates that the use of micrometre-sized SiO_2_ particles is limited. Peng et al. [26] investigated the load-bearing capacity of grease with SiO_2_ NPs between 58 and 648 nm and found that the smallest NPs provided the best tribological performance. However, their study included a long-term ball-on-ring test. Nevertheless, it was concluded consistently with the results presented in this work that lubrication efficiency depends not only on the content and size of the NPs but primarily on the load. Therefore, determining the optimum particle content for a wide range of friction conditions may be impossible.

### 3.2. Surface Topography

**Figure 12:**
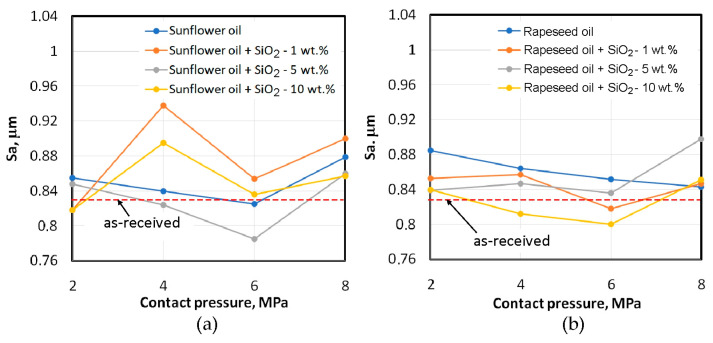
Effect of lubrication conditions on the average roughness Sa for (**a**) sunflower and (**b**) rapeseed oil.

This section presents a discussion of the influence of friction conditions on changes in the surface topography of the sheets. Measuring the surface topography changes enables the identification of the main friction mechanisms (flattening, ploughing) that cause changes in the profile ordinate distribution and thus, the surface load-bearing capacity. In addition, knowledge of the main tribological parameters provides an assessment of the impact of friction on changes in the surface roughness of the finished components. The basic surface roughness parameter Sa, the kurtosis Sku and the skewness Ssk were selected for this analysis. The last two parameters are recommended in the analysis of surface topography changes resulting from lubrication conditions [60,61] and in the analysis of the coefficient of friction of rough surfaces [62]. Figure 12 shows the influence of lubrication conditions on the changes in the average roughness Sa of the samples. For the sunflower oil lubrication conditions, two clear trends can be observed. For the unmodified oil and the oil containing 5 wt.% of SiO_2_, the increase in contact pressure leads to a tendency to reduce the Sa parameter but only up to a contact pressure of 6 MPa (Figure 12a). Above this value, for all lubrication conditions, an increase in the average roughness value occurs in relation to the average roughness of the sheet metal in the as-received state. During friction with oils containing 1 wt.% and 10 wt.% of SiO_2_, after an initial small decrease in the Sa parameter at a contact pressure of 2 MPa, there is a sharp increase at 4 MPa and then a decrease.

**Figure 13:**
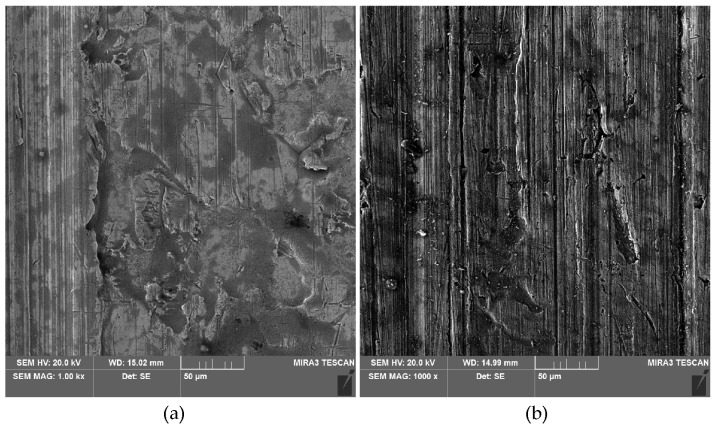
SEM micrographs of the sheet surfaces after the friction tests under the following lubricated conditions: sunflower oil + SiO_2_ (10 wt.%) and contact pressure (**a**) 4 MPa and (**b**) 8 MPa.

During friction with rapeseed oil, similar to sunflower oil, an increase in the Sa parameter was observed after exceeding a contact pressure of 6 MPa but only for the modified oils. This relationship can be explained by the ploughing effect of the hard SiO_2_ particles on the sheet metal surface after exceeding a specific contact pressure value, which in our studies is 6 MPa. Up to this contact pressure value, the general trend is a decrease in the Sa parameter, which corresponds to the flattening phenomenon of the sheet metal surface (Figure 13). During friction with pure rapeseed oil, a continuous decrease in the Sa parameter can be observed with increasing contact pressure between 2 and 6 MPa.

It should be noted that the greatest increase in the Sa parameter in relation to the as-received surface occurred for a contact pressure of 2 MPa (Figure 12b). After the friction process with sunflower oil, the lowest average roughness Sa was observed for the oil containing 5 wt.% of SiO_2_ particles (Figure 12a). In relation to rapeseed oil, the addition of 10 wt.% of SiO_2_ provided a surface with the lowest average roughness (Figure 12b).

**Figure 14:**
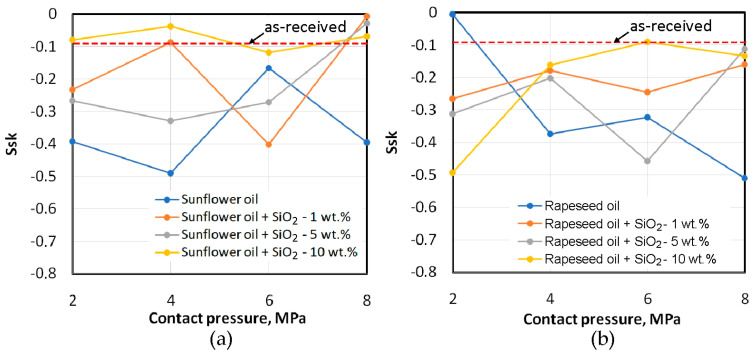
Effect of lubrication conditions on the skewness Ssk for (**a**) sunflower and (**b**) rapeseed oils.

**Figure 15:**
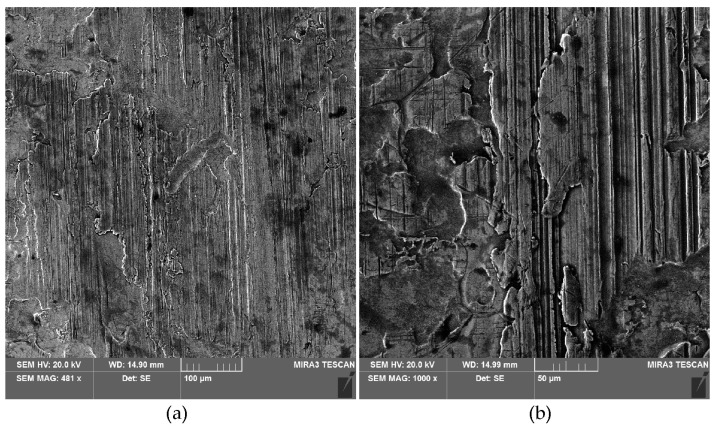
SEM micrographs of the sheet surface after the friction tests under the following lubricated conditions: (**a**) dry friction, contact pressure 8 MPa and (**b**) rape seed oil (unmodified), contact pressure 8 MPa.

The influence of contact pressures and friction conditions on the change of the skewness parameter Ssk of the sheet surface is shown in Figure 14. The negative skewness value indicates the occurrence of relatively deep valleys and flattened summits of the asperities. The addition of 10 wt.% of particles to the sunflower oil provided the smallest change in the skewness compared to the skewness of the as-received sheet surface (Figure 14a). During friction with rapeseed oil at the highest considered contact pressure, the flattening phenomenon (Figure 15b) was at a level similar to that under dry friction conditions (Figure 15a). It can also be seen that, except for one point on the graph, friction with unmodified sunflower oil causes the largest change in the skewness Ssk.

**Figure 16:**
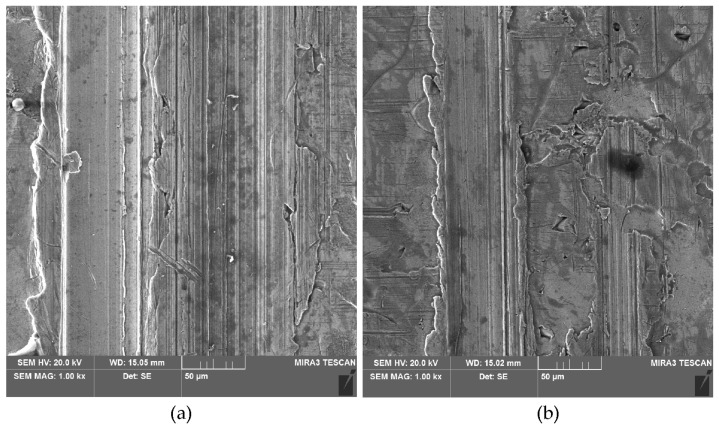
SEM micrographs of the sheet surface after the friction tests under the following lubricated conditions: (**a**) rape seed oil + SiO_2_ (1 wt.%), contact pressure 8 MPa and (**b**) rape seed oil + SiO_2_ (5 wt.%), contact pressure 6 MPa.

The deeper the scratches and grooves on the surface (Figure 16), the more negative the value of the Ssk parameter (Figure 14). Friction with rapeseed oil shows a different character (Figure 14b). Apart from the contact pressure of 2 MPa, again the oil with the addition of 10 wt.% of SiO_2_ can be considered the most neutral in changing the topography of the as-received surface. The clear tendency for the Ssk parameter to decrease with the contact pressure during friction with the participation of pure rapeseed oil (Figure 14b) can be explained by the lack of particles available to separate the rubbing surfaces from metallic contact. For lubrication with sunflower oil, the relation of the effect of the pure oil on the Ssk parameter was different, so the above conclusion can also be related to the physical properties of the oils [63,64].

**Figure 17:**
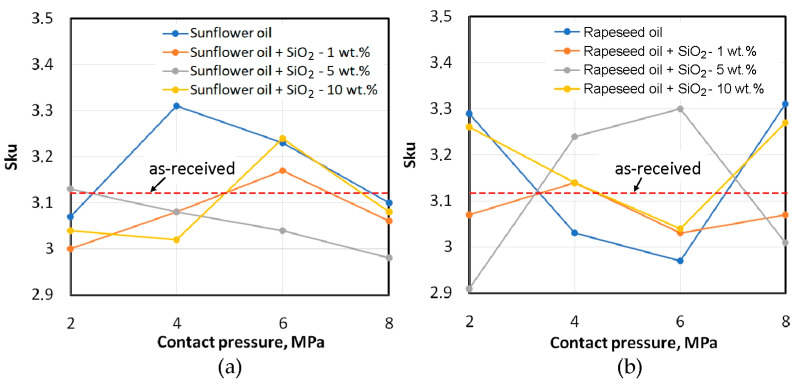
Effect of lubrication conditions on the kurtosis Sku for (**a**) sunflower and (**b**) rapeseed oils.

Kurtosis of the as-received surface greater than 3 (Sku > 3) means that the material ratio curve is bell-shaped, that is, the surface is characterised by shallow valleys and a large number of high peaks [62]. The variations in kurtosis for pure sunflower oil and sunflower oil containing 1 wt.% and 10 wt.% of SiO_2_ particles are similar (Figure 17a). With increasing pressure, kurtosis increases, and after a certain pressure value, kurtosis decreases. Decreasing kurtosis means that the sheet surface is subject to the phenomenon of flattening of the surface asperities. At the highest analysed contact pressure (8 MPa) for the sunflower oil lubrication conditions, a decrease in kurtosis was obtained relative to the as-received surface. At the highest analysed contact pressure, the lubricant was not able to effectively reduce friction. Such conditions occur when there is intensive flattening and roughening of the surface asperities. The least favourable properties from the point of view of the Sku parameter are shown by the sunflower oil with the addition of 5 wt.% of SiO_2_ (Figure 17a), which leads to flattening of the summits of the asperities over the entire range of applied contact pressures. At the same time, the unmodified sunflower oil at a pressure of 4 MPa and the oil modified by the addition of 10 wt.% of SiO_2_ at a pressure of 6 MPa cause the formation of many high peaks and shallow valleys, which correspond to the increase in kurtosis [65]. During lubrication with pure rapeseed oil and oil modified by the addition of 5 wt.% of SiO_2_, the change in the Sku parameter is almost the opposite (Figure 17b).

At low contact pressure in unfavourable conditions, when the effectiveness of the lubricant is too low, the effect of mechanical interactions of the surface asperities may increase. Increasing the contact pressure is unfavourable from the point of view of friction, but in lubricated conditions, the lubricant is subject to greater pressure in closed oil pockets, which is favourable for friction reduction. Meanwhile, the effect of friction with rapeseed oil containing 1 wt.% and 10 wt.% of SiO_2_ on kurtosis is similar to that for friction with pure rapeseed oil. First, there is a tendency to reduce the Sku parameter with increasing contact pressure and then an increase in kurtosis for a contact pressure of 8 MPa (Figure 17b).

The primary purpose of lubrication in deep-drawing processes is to reduce the coefficient of friction. Reducing this reduces the forming forces and allows for greater sheet metal deformations (by reducing the sliding resistance of the sheet metal against the tool surface) [66,67]. The deep-drawing process should have the least possible effect on the change in surface topography [68]. The sheet metal undergoes intentional plastic deformations, which to some extent changes its topography. Appropriate friction conditions should, on the one hand, reduce the coefficient of friction and, on the other, not lead to the phenomenon of deep grooves in the sheet metal surface. The sheet metals used in the automotive industry are characterised by a determined texture [69], which supports the lubrication process through the valleys occurring on the sheet surface. These sheets are characterised by an appropriate texture created in the rolling process with textured rolls. The friction process should have the least possible effect on changing the surface topography of the as-received sheet. The lubricant present in the closed oil pockets is subjected to pressure that limits the metallic contact of the two rubbing surfaces. Additionally, hard SiO_2_ particles interact with the tool and sheet metal surfaces through three mechanisms: polishing, mending and the rolling effect [26,54,57].

Most of the published research results concern the use of NPs to support lubrication efficiency. The concentrations of particles used in the literature are mainly between 0.01 and 5 wt.%. Considering that cold-rolled steel sheets are characterised by relatively high roughness, this article proposes the use of micrometre-sized NPs to modify the oils intended for the deep-drawing process. On the one hand, vegetable oil-based lubricants can replace synthetic oils, contributing to the development of a sustainable industry [70], and on the other hand, SiO_2_ particles contribute to a significant reduction in the coefficient of friction. This paper also points out that the optimal content of particles applies only to narrow friction conditions. A change in contact pressure or a different type of base oil forces the search for optimal properties and contents of the lubricant [71]. The results of the study confirmed that the presence of hard particles in the base oil improves the lubrication efficiency and ensures less change in the surface topography compared to unmodified oil lubrication conditions. In this respect, the results of the study are similar to those of Bao et al. [72] and Tao et al. [73]. It was also shown that at some pressure values, increasing the particle weight content to 10% gives better results than the maximum concentration of 5 wt.% used in the literature [74]. The greatest reduction in the friction coefficient value with the modified oil was about 77% (Figure 10). Based on the literature analysis, Singh et al. [75] achieved the highest reduction in the CoF of 91.6%.

## 4. Conclusions

Based on the conducted research on the potential use of biolubricants in the sheet metal forming of dual-phase HCT600X+Z steel sheets, the following conclusions can be drawn:

- For the entire range of pressures analysed, unmodified sunflower oil showed lower efficiency in reducing the CoF compared to unmodified rapeseed oil.
- The highest lubrication efficiency of unmodified oils was observed at the lowest analysed pressure of 2 MPa: 26.7% for sunflower oil and 37.7% for rapeseed oil.
- At the lowest analysed contact pressure, the effect of the addition of SiO_2_ particles on the CoF is not clear. Perhaps at this contact pressure, the surface roughness did not change much, and local closed oil pockets were not created. To ensure proper lubrication, it is necessary to generate an appropriate lubricant pressure. In the contact pressure range of 4–8 MPa, biolubricants ensured a reduction in the CoF relative to dry friction conditions.
- In the pressure range of 4–8 MPa, the lubricants with 5 wt.% and 10 wt.% of SiO_2_ particles were more effective in reducing friction than the biolubricant with addition of 1 wt.% of SiO_2_. These lubricants reduced the coefficient of friction to almost the same extent, between 26.5% (6 MPa) and 34.9% (4 MPa), relative to dry friction conditions.
- Lubrication with unmodified oil and oil containing 5 wt.% of SiO_2_ leads to a tendency to reduce the Sa parameter with increasing contact pressure but only up to a contact pressure of 6 MPa. After exceeding this value for all lubrication conditions, as a result of intensive flattening and roughening, an increase in the Sa parameter value occurs relative to the sheet metal in the as-received state.
- The lowest average roughness was observed for lubrication with sunflower oil containing particles at a concentration of 5 wt.%. For rapeseed oil, the addition of 10 wt.% of SiO_2_ provided a sheet surface with the lowest average roughness.
- The negative value of the skewness of the sheets before and after the friction process indicates the occurrence of relatively deep valleys and flattened peaks of the asperities on the surface. The addition of 10 wt.% of particles to sunflower oil provided the smallest change in skewness compared to the surface of the as-received sheet.
- With the increase in contact pressure, kurtosis increases, and after a certain pressure value, depending on the type of lubricant, kurtosis decreases. This means that the sheet metal surface is subject to the phenomenon of flattening of the surface asperities. At the highest analysed contact pressure for all lubrication conditions with sunflower oil, a decrease in kurtosis was obtained relative to the as-received surface. The same relationship was confirmed for rapeseed oil with the addition of 1 and 5 wt.% of SiO_2_.

## Figures and Tables

**Table 1:** Average mechanical properties of HCT600X+Z steel sheet.

Young’s Modulus, GPa	Yield Stress, MPa	Ultimate Tensile Strength, MPa	Elongation A_80_, %	Strain Hardening Exponent
199	420	662	23.62	0.188

**Table 2:** Experimental plan.

Experiment Number	Contact Pressure, MPa	Friction Conditions
1	2	Dry friction
2	2	Sunflower oil
3	2	Sunflower oil + SiO_2_ (1 wt.%)
4	2	Sunflower oil + SiO_2_ (5 wt.%)
5	2	Sunflower oil + SiO_2_ (10 wt.%)
6	2	Rapeseed oil
7	2	Rapeseed oil + SiO_2_ (1 wt.%)
8	2	Rapeseed oil + SiO_2_ (5 wt.%)
9	2	Rapeseed oil + SiO_2_ (10 wt.%)
10	4	Dry friction
11	4	Sunflower oil
12	4	Sunflower oil + SiO_2_ (1 wt.%)
13	4	Sunflower oil + SiO_2_ (5 wt.%)
14	4	Sunflower oil + SiO_2_ (10 wt.%)
15	4	Rapeseed oil
16	4	Rapeseed oil + SiO_2_ (1 wt.%)
17	4	Rapeseed oil + SiO_2_ (5 wt.%)
18	4	Rapeseed oil + SiO_2_ (10 wt.%)
19	6	Dry friction
20	6	Sunflower oil
21	6	Sunflower oil + SiO_2_ (1 wt.%)
22	6	Sunflower oil + SiO_2_ (5 wt.%)
23	6	Sunflower oil + SiO_2_ (10 wt.%)
24	6	Rapeseed oil
25	6	Rapeseed oil + SiO_2_ (1 wt.%)
26	6	Rapeseed oil + SiO_2_ (5 wt.%)
27	6	Rapeseed oil + SiO_2_ (10 wt.%)
28	8	Dry friction
29	8	Sunflower oil
30	8	Sunflower oil + SiO_2_ (1 wt.%)
31	8	Sunflower oil + SiO_2_ (5 wt.%)
32	8	Sunflower oil + SiO_2_ (10 wt.%)
33	8	Rapeseed oil
34	8	Rapeseed oil + SiO_2_ (1 wt.%)
35	8	Rapeseed oil + SiO_2_ (5 wt.%)
36	8	Rapeseed oil + SiO_2_ (10 wt.%)

## Data Availability

The original contributions presented in the study are included in the article, further inquiries can be directed to the corresponding author.
